# Signaling into the nucleus through the importin 7 pathway

**DOI:** 10.1016/j.jbc.2026.113382

**Published:** 2026-07-30

**Authors:** Oksana Palchevska, Ying-Hui Ko, Gino Cingolani

**Affiliations:** Department of Biochemistry and Molecular Genetics, University of Alabama at Birmingham, Birmingham, Alabama, USA

**Keywords:** nucleocytoplasmic transport, NPC, NLS, importin 7, importin β, β-karyopherins, cytokine signaling

## Abstract

Importin 7 (IPO7) is a nuclear transport receptor of the β-karyopherin family that mediates the translocation of a broad spectrum of macromolecules, commonly referred to as cargoes. Discovered nearly three decades ago, IPO7 was initially identified as an import receptor for constitutive cellular cargoes, including histone H1 and ribosomal proteins, and was shown to function synergistically and partially redundantly with canonical receptors such as importin β1 and karyopherin β2. Over the past 15 years, however, accumulating evidence has established IPO7 as an important mediator of signal-dependent nuclear trafficking in response to extracellular stimuli, including cytokines, growth factors, and cellular stress. Thus, IPO7 has emerged as a versatile nuclear transport receptor that couples extracellular signaling to dynamic changes in nuclear composition and gene expression. Mechanistically, many IPO7 cargoes lack classical nuclear localization signals and instead contain noncanonical targeting motifs that directly engage IPO7. In several cases, phosphorylation-dependent conformational changes expose these motifs, promoting IPO7 binding and translocation through the nuclear pore complex. The expanding repertoire of IPO7 cargoes, including ERK, SMAD3, EGR1, GLI1, the glucocorticoid receptor, HIF-1α, YAP1, and RUNX2, highlights its prominent role at the interface of signaling and transcriptional control. Consistent with these functions, dysregulation of IPO7-mediated transport has been implicated in cancer, hypoxia, and other pathological states. Beyond cellular signaling, IPO7 also contributes to the nuclear trafficking of viral genomes and proteins. Here, we review the molecular mechanisms of IPO7-dependent nuclear import, emerging principles of cargo recognition, and pathways that regulate IPO7 activity during cellular signaling.

Importin 7 (IPO7) is an evolutionarily conserved nuclear import receptor that transports a remarkably diverse repertoire of macromolecules into the nucleus. Initially regarded as a housekeeping transport factor, IPO7 has since emerged as a versatile import receptor for stimulus-induced cargoes. Through the nuclear import of proteins involved in chromatin organization, ribosome biogenesis, signal transduction, gene expression, and antiviral responses, IPO7 plays broad and essential roles in cell physiology. More recently, dysregulation of IPO7-dependent transport has been implicated in viral infection and cancer, underscoring its growing biological and biomedical significance and highlighting IPO7 as an emerging therapeutic target. In this review, we examine IPO7 from a different perspective: not simply as a nuclear import receptor, but as a signaling hub that couples extracellular cues to dynamic changes in nuclear composition and gene expression.

### Constitutive *versus* stimulus-induced nuclear transport

The compartmentalization of translation in the cytoplasm and transcription in the nucleus forces eukaryotic cells to constantly exchange macromolecules between the nucleus and cytoplasm to maintain basic cellular functions (1). Nucleocytoplasmic transport occurs through the nuclear pore complex (NPC) (2, 3), a massive proteinaceous structure embedded in the nuclear envelope that functions as a key gatekeeper to the nucleus and a semipermeable filter, preventing the free diffusion of larger macromolecules into the nucleus. Constitutive transport through the NPC, mediated by β-karyopherins and the Ras-related GTPase Ran (1, 4, 5, 6, 7), represents a fundamental housekeeping activity in nucleated cells.

The first discovered and most extensively studied pathway for protein import from the cytoplasm to the nucleus, known as the classical import pathway, relies on classical nuclear localization signals (cNLSs), which are recognized by importin α within the importin α/β1 heterodimer. In this pathway, importin α serves as an adapter (8, 9) for the transport receptor importin β1 (10, 11). Atomic-level information is available for importin β1 recognition of the importin-β-binding (IBB) domain of importin α (12, 13) (Table 1), used by cargoes bearing a cNLS (14), as well as nonclassical cargoes that bypass the adapter and directly bind to importin β1, such as SREBP-2 (15) and PTHrP (16). RanGTP binds importin β1 with high affinity in the nucleus, or possibly at the nucleoplasmic face of the NPC, inducing a conformational change that displaces both the importin α–NLS-cargo complex and the phenylalanine-glycine (FG) repeats of nucleoporins lining the interior of the NPC (17).

**Table 1:** List of 20 human β-karyopherins

Clade	Protein name	HGNC Symbol	Common protein names	Yeast homolog	Gene aliases	UniProt accession	PDB/AF entry
1a	Importin β1	KPNB1	Impβ1, Importin β, Karyopherin β1, p97, PTAC97	Kap95	*KPNB1*	Q14974	PDB-1QGK; >50 entries
1a	Transportin 1	TNPO1	Transportin, Importin β2, Impβ2, Karyopherin β2, Kapβ2	Kap104	*TNPO1*, *KPNB2*	Q92973	PDB-1QBK; >50 entries
1a	Transportin 2	TNPO2	Importin β2b, Impβ2b, Karyopherin β2b, Kapβ2b	Kap104	*TNPO2*	O14787	AF-O14787-F1
1b	Importin 4	IPO4	RanBP4	Yrb4/Kap123	*IPO4*, *IMP4B*, *RANBP4*	Q8TEX9	PDB-5XBK
1b	Importin 5	IPO5	RanBP5	Pse1/Kap121	*IPO5*, *KPNB3*, *RANBP5*	O00410	PDB-6XTE
1b	Ran-binding protein 6	RanBP6	-	Kap104	*RANBP6*	O60518	AF-O60518-F1
2a	Importin 11	IPO11	RanBP11, Imp11	Kap120	*IPO11*, *RANBP11*	Q9UI26	AF-Q9UI26-F1
2a	Importin 9	IPO9	RanBP9, Imp9	Kap114	*IPO9*, *IMP9*, *KIAA1192*, *RANBP9*	Q96P70	AF-Q96P70-F1
2b	Exportin 2	XPO2	CAS	Cse1	*CSE1L, CAS, XPO2*	P55060	PDB-1WA5
2b	**Importin 7**	**IPO7**	**RanBP7, Imp7**	**Nmd5/Kap119**	***IPO7*, *RANBP7***	O95373	**PDB-6N88; 9QF0**
2b	Importin 8	IPO8	RanBP8, Imp8	Sxm1/Kap108	*IPO8*, *RANBP8*	O15397	AF-O15397-F1
3a	Exportin 1	XPO1	CRM1	Crm1	*XPO1*, *CRM1*	O14980	PDB-3GJX; >50 entries
3a	Exportin 5	XPO5	RanBP21, Exp5	Msn5	*XPO5, KIAA1291, RANBP21*	Q9HAV4	PDB-3A6P
3b	Exportin 4	XPO4	Exp4	N/A	*XPO4*, *KIAA1721*	Q9C0E2	AF-Q9C0E2-F1
3b	Exportin 7	XPO7	RanBP16, Exp7	N/A	*XPO7*, *KIAA0745*, *RANBP16*	Q9UIA9	AF-Q9UIA9-F1
3b	Importin 3	IPO3	RanBP17	N/A	*RANBP17*	Q9H2T7	AF-Q9H2T7-F1
3c	Exportin-T	XPOT	Exportin (tRNA), XPO3	Los1	*XPOT*	O43592	PDB-3ICQ
3c	Exportin 6	XPO6	RanBP20, Exp6	N/A	*XPO6*, *KIAA0370*, *RANBP20*	Q96QU8	AF-Q96QU8-F1
3d	Importin 13	IPO13	RanBP13, Imp13	Pdr6/Kap122	*IPO13*, *KIAA0724*, *RANBP13*	O94829	PDB-3ZJY
3d	Transportin 3	TNPO3	Importin β12, Transportin-SR2	Mtr10	*TNPO3*, *IPO12*	Q9Y5L0	AF-Q9Y5L0-F1

Members are grouped according to the phylogenetic classification shown in Figure 1. IPO7 is highlighted in bold.

Abbreviations: HGNC, HUGO Gene Nomenclature Committee; AF, AlphaFold Protein Structure Database; PDB, Protein Data Bank.

However, beyond constitutive nuclear import, intracellular signaling pathways transmit signals from various extracellular stimuli to their cytosolic targets, triggering biological responses within the nucleus and leading to cellular events such as proliferation, differentiation, cell death, and migration (18). Thus, in addition to constitutive nuclear import pathways, cells have evolved mechanisms to rapidly transduce signals from outside the cell directly into the nucleus, typically to access chromatin. This is known as stimulus-induced nuclear import (19). Signal transmission from the cytoplasm to the nucleus often requires the nuclear translocation of one or more β-karyopherins. The mechanisms by which these components reach the nucleus upon stimulation have only been partially understood, and only for a few signaling pathways (20, 21, 22, 23).

### IPO7 is a member of the β-karyopherin family

The bulk of active nuclear transport is carried out by soluble transport receptors of the β-karyopherin family. The human genome encodes twenty β-karyopherins (24, 25, 26, 27) that share relatively low sequence similarity but adopt a conserved superhelical architecture built from tandem Huntingtin, Elongation factor 3, protein phosphatase 2A, and TOR1 (HEAT) repeats (28). β-karyopherins were initially identified by the presence of an N-terminal RanGTP-binding region localized within the first 10 HEAT repeats (26, 29). This region also contains binding sites for FG-repeats (17, 30, 31) exposed by nucleoporins, which β-karyopherins bind to with different degrees of affinity during transport (32, 33). Phylogenetic analysis of the twenty human β-karyopherins segregates these proteins into three evolutionarily related groups that share a common ancestor, or clades (25, 34), named after their representative members: importin β1/KPNB1 (clade 1), IPO7 (clade 2), and CRM1/XPO1 (clade 3) (Fig. 1, Table 1).

**Figure 1:**
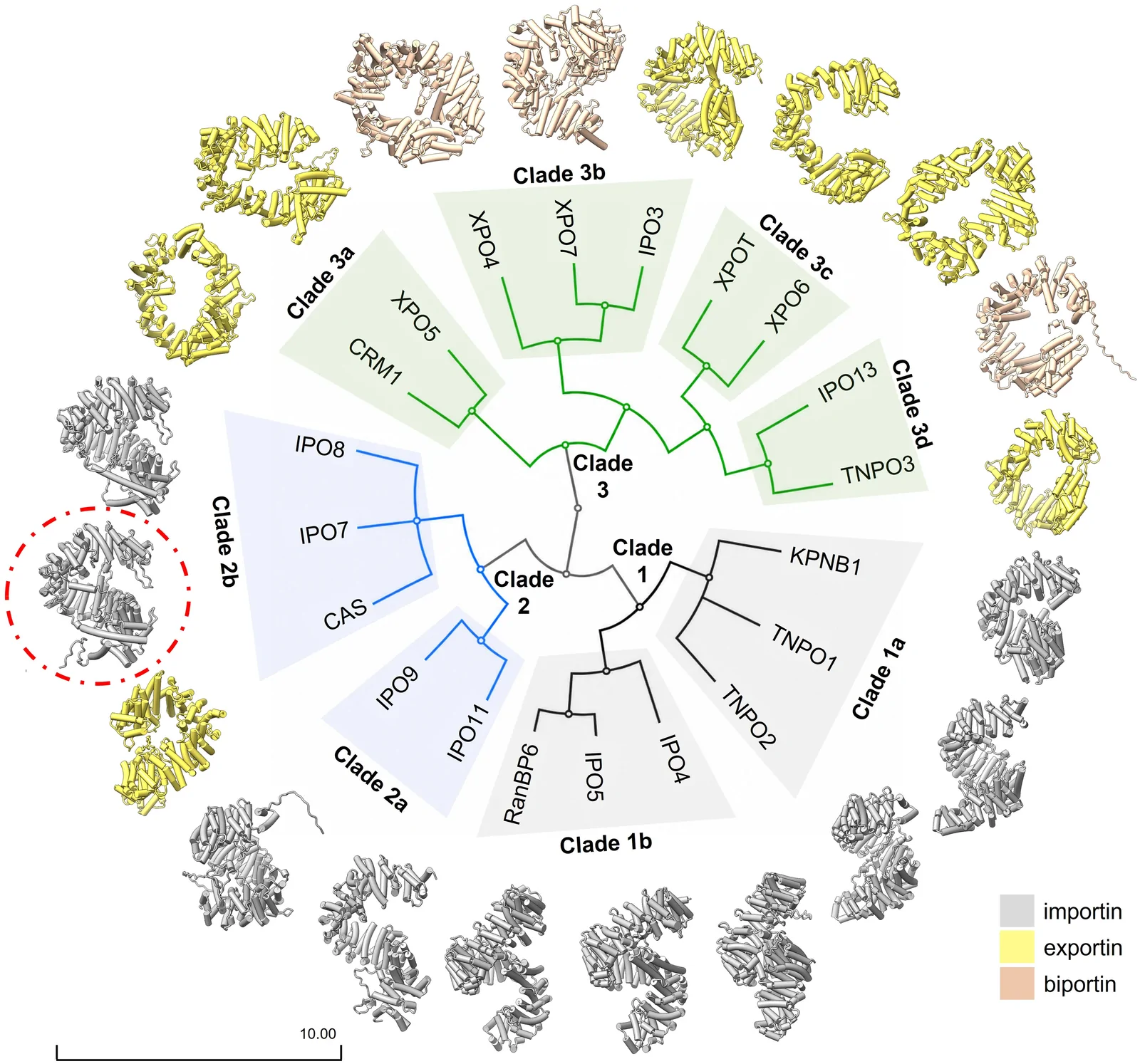
**Phylogenetic organization of the human β-karyopherin family.** Radial phylogram of the 20 human β-karyopherins generated from a multiple sequence alignment of their amino acid sequences (Table 1). Three major evolutionary lineages are evident: clades 1 (*black*), 2 (*blue*), and 3 (*green*). AlphaFold-predicted (155) structures of all human β-karyopherins are displayed around the phylogram and colored according to transport function: importins (*gray*), exportins (*yellow*), and biportins (*orange*). IPO7 (*red dashed circle*) clusters with IPO8 and CAS within clade 2b, reflecting their close evolutionary relationship. Branch lengths are proportional to evolutionary distance, and the scale bar represents the expected number of amino acid substitutions per site. Multiple sequence alignment was performed using ClustalW (156), and phylogenetic analysis was carried out using the Simple Phylogeny tool at EMBL-EBI employing the ClustalW2 neighbor-joining algorithm. AlphaFold models were rendered using ChimeraX (157). CAS, cellular apoptosis susceptibility protein (exportin 2); CRM1, chromosome region maintenance 1 (exportin 1); HEAT, huntingtin, elongation factor 3, protein phosphatase 2A, and TOR1; IPO, importin; KPNB1, karyopherin subunit β1 (importin β1); TNPO, transportin; XPO, exportin.

Clade one comprises exclusively nuclear import receptors and is divided into two subclades (Fig. 1). Clade 1a includes the universal import receptor importin β1 (KPNB1) (35, 36), which mediates cNLS import, transportin 1 (TNPO1), also known as karyopherin β2 (Kapβ2) (37), which mediates the nuclear import of proteins containing a proline-tyrosine nuclear localization signal (PY-NLS) (38), and transportin 2 (39). In contrast, Clade 1b contains IPO4, a nuclear import receptor for the vitamin D receptor (29), IPO5 (40), and Ran-binding protein 6 (32), three evolutionarily related and functionally specialized import receptors.

Clade two also segregates into two subgroups but comprises both import and export receptors (Fig. 1). Clade 2a contains IPO11 (41), best known for the selective import of ubiquitin-conjugating enzymes, and IPO9, which has been implicated in the import of RNP-associated proteins (42). Clade 2b includes exportin 2 (XPO2), also known as CAS, which recycles importin α to the cytoplasm (43), together with the closely related import receptors IPO7 (40), the focus of this review, and IPO8 (44).

Clade three is the largest and most functionally diverse branch of the β-karyopherin family, comprising nine members organized into four subclades (Fig. 1). Clade 3a contains the major nuclear export receptor exportin 1 (XPO1), also known as CRM1 (45, 46, 47), together with the pre-miRNA export receptor XPO5 (48). Clade 3b includes XPO7, previously known as RanBP16 (32, 49), and XPO4 (50), both bidirectional transport receptors (biportins), as well as IPO3, implicated in the nuclear transport of transcription factors and viral RNA, although its physiological cargo repertoire remains largely undefined (51). Clade 3c comprises the tRNA export receptor exportin-T (52, 53) and XPO6 (54), whereas Clade 3d contains the biportin IPO13 (55) and TNPO3, also known as transportin-SR2, a specialized import receptor for serine/arginine-rich (SR) proteins (56).

Together, these phylogenetic relationships highlight the evolutionary diversification of β-karyopherins into structurally related yet functionally specialized nuclear transport receptors that can function as import receptors, export receptors, or both (termed biportins) (Fig. 1).

### HEAT repeats generate functionally polarized surfaces

Despite substantial sequence divergence within individual clades, all β-karyopherins retain a conserved HEAT-repeat organization that folds into flexible solenoid-like scaffolds, providing extended protein–protein interaction surfaces that support cargo recognition and interactions with the NPC and nuclear transport machinery (28). Each HEAT repeat consists of two antiparallel α-helices (Fig. 2*A*), and successive repeats stack together to form a flexible solenoid structure with two functionally distinct surfaces: a concave inner surface that typically mediates interactions with cargoes and RanGTP, and a convex outer surface that binds FG-repeat nucleoporins (Fig. 2*B*).

**Figure 2:**
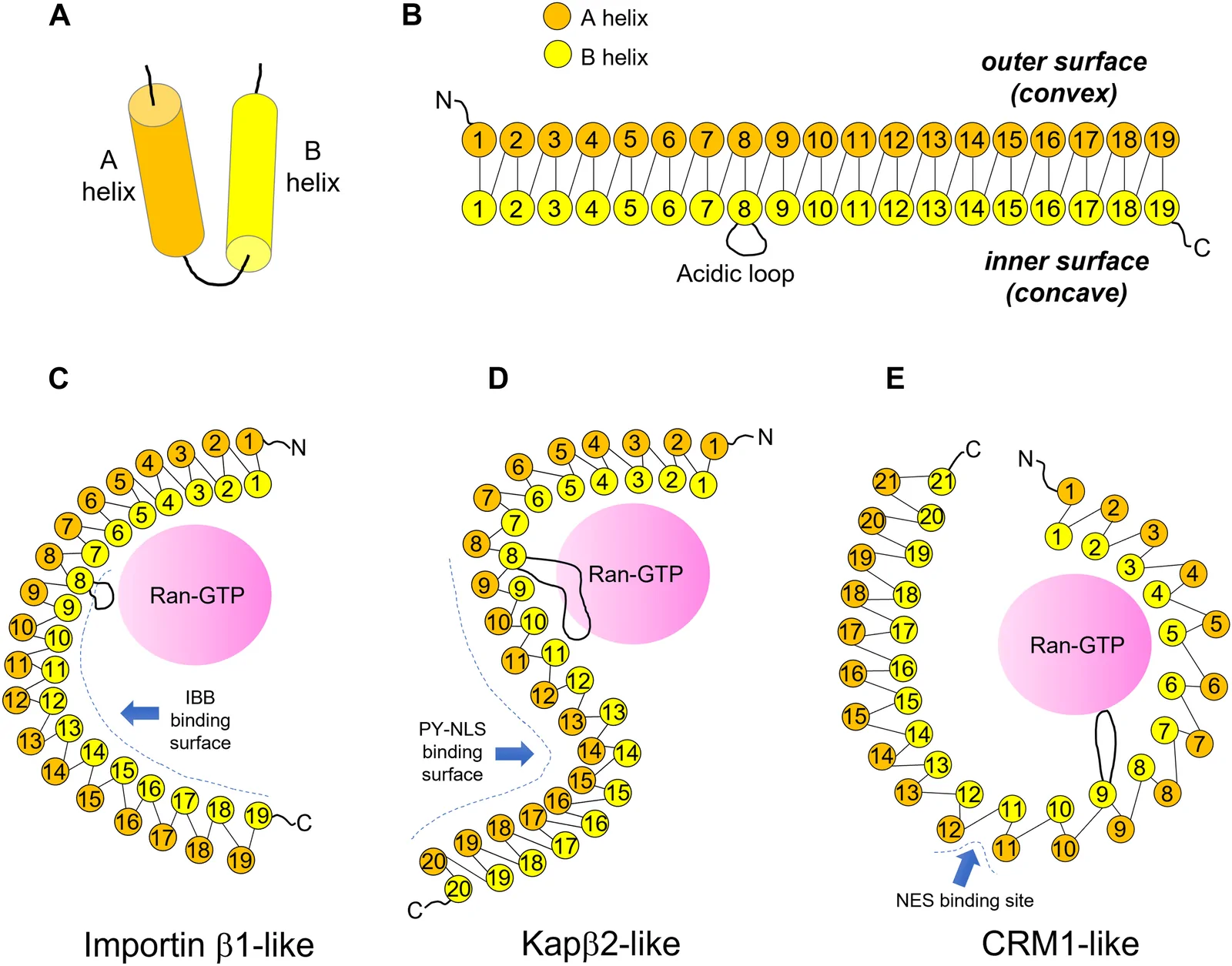
**Conserved HEAT-repeat topology of β-karyopherins.***A*, schematic representation of a canonical HEAT repeat, consisting of an outer A helix and an inner B helix. *B*, linear topology of the representative importin β1, showing its 19 HEAT repeats and the acidic loop inserted within HEAT repeat 8, between the A and B helices. *Orange* and *yellow circles* denote A and B helices, respectively. *C*, *D*, and *E*, functional organization of representative β-karyopherins. *C*, importin β1 binds RanGTP through its N-terminal HEAT repeats and most cargoes through an extended C-terminal surface. *D*, Kapβ2 binds RanGTP through its N-terminal arch and PY-NLS cargoes through its C-terminal arch; the H8 acidic loop couples RanGTP binding to cargo release. *E*, CRM1 binds RanGTP through its N-terminal HEAT repeats and H9 acidic loop, whereas leucine-rich NES cargoes bind a hydrophobic groove located primarily between HEAT repeats 11 and 12. HEAT, huntingtin, elongation factor 3, protein phosphatase 2A, and TOR1; IBB, importin β-binding domain; Kapβ2, karyopherin β2; TNPO1, transportin-1; NES, nuclear export signal; PY-NLS, proline-tyrosine nuclear localization signal; RanGTP, GTP-bound Ran GTPase.

Decades of structural and biophysical studies have established that β-karyopherins are highly deformable solenoids whose HEAT-repeat architecture confers remarkable conformational plasticity (57, 58, 59), enabling cargo recognition, Ran-dependent allostery, and translocation through the FG-rich environment of the NPC (17). The three best characterized β-karyopherins, importin β1 and Kapβ2, both import receptors, and CRM1, an export factor, have been visualized in complex with several binding partners using both X-ray crystallography and cryo-EM methods (Table 1). Three predominant HEAT-solenoid conformations have been reported: a C-shaped conformation, exemplified by importin β1 bound to IBB or RanGTP (13, 17) (Fig. 2*C*); an S-shaped conformation, like in Kapβ2 bound to PY-NLS or RanGTP (38, 60) (Fig. 2*D*); and, finally, a closed O-shaped conformation typical of CRM1 bound to RanGTP and cargos bearing a nuclear export signal (61) (Fig. 2*E*). Based on AlphaFold predictions and a low-resolution cryo-EM structure (62) (Table 1), IPO7 comprises 20 HEAT repeats and adopts an S-shaped architecture that more closely resembles Kapβ2 than importin β1.

Overall, the remarkable structural adaptability of β-karyopherins likely provided the evolutionary framework for diversification of the β-karyopherin family while preserving a common mechanistic strategy for cargo transport across the NPC.

## Molecular functions of IPO7

IPO7 is a ∼120 kDa member of the β-karyopherin family (circled in red in Fig. 1 and Table 1), composed of 20 HEAT repeats and encoded by the IPO7 gene on chromosome 11p15.4 (NCBI Gene ID: 10527). IPO7 was first identified in 1997 in the Görlich laboratory as a RanGTP-binding protein and was originally termed Ran-binding protein 7 (RanBP7) (29). However, unlike other RanBPs, IPO7 functions as an import receptor. Early studies reported that IPO7 associates with the IBB domain of importin α through importin β1 rather than by direct binding (29). Subsequent work identified the importin β1–IPO7 heterodimer as a functional nuclear import receptor for histone H1 (63). Consistent with this, IPO7 does not recognize cNLSs, supporting its role in the nuclear import of nonclassical cargoes. Over the last 3 decades, IPO7 has emerged as a mediator of both constitutive nuclear import, required for cellular homeostasis, and stimulus-induced import triggered by extracellular signaling. In addition, posttranslational modifications, such as phosphorylation and SUMOylation (64, 65) in IPO7 or its cargo proteins, have been demonstrated and are likely important determinants of cargo-specific, high-affinity binding.

IPO7 is evolutionarily conserved across metazoans and belongs to an ancient β-karyopherin clade whose members are present throughout eukaryotes, consistent with a fundamental role in nucleocytoplasmic transport. In budding yeast, the β-karyopherins Pse1/Kap121 and Nmd5/Kap119 represent the closest functional counterparts of the mammalian IPO7/IPO8 lineage (Table 1), with Pse1/Kap121 being essential for viability (66, 67). Similarly, depletion of the IPO7 homolog in *Drosophila melanogaster* is lethal (68). Expression profiling of the human β-karyopherin family demonstrated that IPO7 is ubiquitously expressed across diverse human tissues, supporting its role as a broadly utilized nuclear transport receptor involved in multiple cellular pathways (34).

IPO7 levels are regulated at both transcriptional and posttranslational levels by multiple signaling pathways. Sphingosine directly binds several β-karyopherins, including IPO7, inducing conformational changes that inhibit their nuclear import activity (69). In myofibroblasts, transforming growth factor β (TGF-β) elevates IPO7 protein levels in a p38 mitogen-activated protein kinase (MAPK)-dependent manner. Consistent with its role as a housekeeping factor, KO of IPO7 in mammalian cells is lethal (70). Although no monogenic human disorder has yet been directly attributed to IPO7 deficiency, elevated IPO7 expression at both the mRNA and protein levels has been reported in several cancer models, and IPO7 has been implicated in diverse biological processes, including dental and cartilage development (65, 71, 72, 73, 74), inflammation (75), and neural ectoderm differentiation from mouse embryonic stem cells in cooperation with XPO4 (76).

### Housekeeping function of IPO7 in constitutive nuclear import

**Figure 3:**
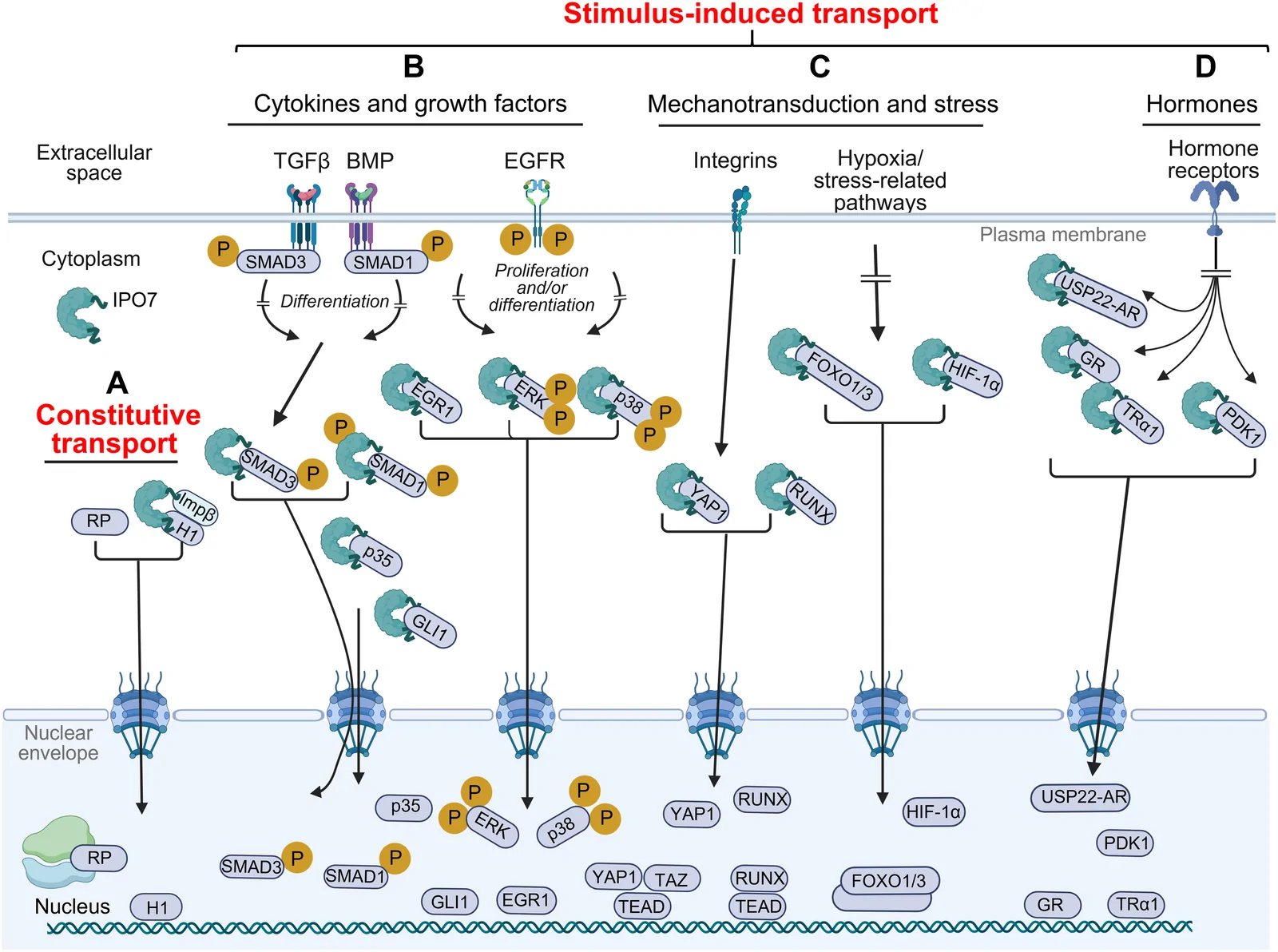
**IPO7-dependent nuclear import pathways.** IPO7 supports both constitutive and stimulus-induced nuclear transport. *A*, constitutive nuclear import of histone H1 and ribosomal proteins. *B*, *C*, and *D*, stimulus-induced nuclear transport pathways include. *B*, cytokine- and growth factor-dependent nuclear import pathways downstream of TGF-β/BMP and EGFR signaling; *C*, mechanotransduction- and oxidative stress-dependent nuclear import pathways; and *D*, hormone-dependent nuclear import pathways. Representative cargoes and signaling pathways are shown. Phosphorylation events are indicated by “P”. The image was created with BioRender.com. AR, androgen receptor; BMP, bone morphogenetic protein; EGR1, early growth response protein 1; EGFR, epidermal growth factor receptor; ERK, extracellular signal-regulated kinase; FOXO1/3, forkhead box O1/3; GLI1, GLI family zinc finger 1; GR, glucocorticoid receptor; H1, histone H1; HIF-1α, hypoxia-inducible factor 1α; Impβ, importin β1; P, phosphate (phosphorylation); PDK1, 3-phosphoinositide-dependent protein kinase 1; p38, p38 mitogen-activated protein kinase; p35, cyclin-dependent kinase 5 activator 1; RP, ribosomal proteins; RUNX, Runt-related transcription factor; SMAD, mothers against decapentaplegic homolog; TAZ, transcriptional coactivator with PDZ-binding motif; TEAD, TEA domain transcription factor; TGF-β, transforming growth factor β; TRα1, thyroid hormone receptor α1; USP22, ubiquitin-specific peptidase 22; YAP, Yes-associated protein 1.

IPO7 functions either as an independent import receptor for ribosomal proteins or together with importin β1 as a heterodimeric import receptor for histone H1, although it remains unclear whether IPO7 acts as an adapter or a coreceptor in the latter complex (63). This dual mode of action allows IPO7 to contribute to both constitutive nuclear import and broader aspects of protein homeostasis (77, 78), depending on the cargo and cellular context. The earliest characterized function of the IPO7 pathway involves the translocation of housekeeping cargoes, such as histone H1 (63) and ribosomal proteins (*e.g.*, rpL4 and rpL6) (Fig. 3*A*) (40, 79). These cargoes bind directly to IPO7 and importin β1, without the adapter importin α (63, 68, 80). The site on ribosomal proteins that binds nuclear receptors was first identified and termed BIB (40), as it is the same site that associates with IPO5 and IPO7, transportin, and importin β1. This sequence is very basic and appears to be predominantly random coil, unlike the IBB domain (12), and is more complex than a cNLS (14).

For histone H1, a basic sequence that resembles a cNLS has been mapped and is recognized by a heterodimer of IPO7–importin β1 (81). A low-resolution (6–8 Å) cryo-EM reconstruction of importin β1–IPO7 bound to H1 has been reported (80). The *pseudo*-atomic model derived from this low-resolution map suggests that histone H1 binds primarily to importin β1 rather than IPO7, partially contradicting previous biochemical data (63). A recent reanalysis of the same cryo-EM density (62) suggested an opposite organization, with IPO7 directly involved in H1 binding, while associated with importin β1, as initially suggested (63). In the absence of a higher-resolution structure of the complex, the role and extent of IPO7's direct receptor function for H1 remain unclear. While these constitutive functions established IPO7 as a housekeeping import receptor, subsequent work revealed that many of its best-characterized functions involve stimulus-induced nuclear import.

### Role of IPO7 in stimulated nuclear import

A broad and expanding set of signaling proteins relies on IPO7. Over the past 15 years, IPO7 has emerged as a key mediator of stimulus-induced nuclear import in response to cytokines, growth factors, and cellular stress (19) (Fig. 3, *B*–*D*). This pathway is distinct from constitutive nucleocytoplasmic shuttling and represents a regulated mechanism that couples extracellular signals to nuclear responses. IPO7-dependent import controls processes such as proliferation (82, 83), stress responses (84), and, when dysregulated, oncogenesis (82, 83, 84, 85, 86). Many IPO7 cargoes lack canonical NLSs and instead contain nonclassical nuclear translocation signals (NTSs) that mediate direct binding to IPO7 (87). In resting cells, these cargoes are retained in the cytoplasm through interactions with anchoring proteins. Upon stimulation, phosphorylation-dependent release exposes the NTS, enabling IPO7 binding and transport through the NPC. RanGTP then promotes cargo release in the nucleus, while IPO7 is recycled (88).

The number of NTS-containing cargoes continues to grow: NTS motifs have been identified in signaling proteins, including the signal transducer mothers against decapentaplegic homolog 3 (SMAD3), the kinases extracellular signal-regulated kinase 1/2 (ERK1/2) (89), the MAPKs 1 and 2 (MEK1/2) (88), the transcription factors early growth response protein 1 (EGR1) (90) and GLI family zinc finger 1 (GLI1) (91). Putative NTSs have been proposed in Notch1 (92), SYK (93), PYK2 (94), SIRT2 (95), Drosha (96), Yes-associated protein 1 (YAP1) (97), Runt-related transcription factor 2 (RUNX2) (72), hypoxia-inducible factor 1α (HIF-1α) (98), and glucocorticoid receptor (GR) (99, 100), although there is insufficient experimental evidence to define a consensus motif and functionally validate its role in nuclear transport. Collectively, these findings suggest that stimulus-induced NTS motifs broadly regulate nuclear import of signaling proteins, with IPO7 serving as an important mediator. However, not all putative NTS cargoes depend exclusively on IPO7. For example, nuclear import of p35 involves importin β1, IPO5, and IPO7 (101) *via* a classical NLS, highlighting redundancy between pathways. Thus, NTS-mediated import likely represents an additional regulatory layer that selectively engages IPO7 under specific conditions.

### Classification of stimuli that activate the IPO7 pathway

The stimuli that trigger IPO7-dependent transport are diverse and include cytokines and growth factors, through the TGF-β signaling and epidermal growth factor receptor/12-O-tetradecanoylphorbol-13-acetate signaling pathways (102) (Fig. 3*B*); integrin signaling and mechanotransduction by which cells convert mechanical forces into biochemical signals (Fig. 3*C*), as well as hormones (Fig. 3*D*). Below, we list critical signaling molecules translocated into the nucleus by IPO7 and sort them by the type of extracellular stimulus that triggers IPO7-mediated nuclear import.

#### a. Cytokines and growth factors

Many cytokines and growth factors signal through the nuclear accumulation of activated transcription factors and protein kinases. IPO7 has emerged as an important mediator of these pathways, linking extracellular signaling to changes in nuclear gene expression (Fig. 3*B*). The TGF-β-responsive transcription factor SMAD3 undergoes IPO7-dependent nuclear import (103, 104). Although it contains an NLS-like sequence, this is insufficient for classical import, and translocation is mediated by IPO7 and IPO8, as with SMAD1 (105, 106). SMAD3 contains a Ser-Pro-Ser-Gln motif that promotes binding to IPO7 upon phosphorylation (88, 107). RanGTP dissociates the IPO7–SMAD3 complex in the nucleus. Redundancy exists, as other karyopherins and nucleoporin-mediated pathways can also contribute to SMAD3 import (103, 108). Similarly, ERK1/2 are prototypical IPO7-dependent cargoes that undergo stimulus-induced nuclear translocation. In resting cells, ERK1/2 are retained in the cytoplasm by anchoring proteins (109, 110). Upon stimulation, phosphorylation of the ERK Thr-Glu-Tyr activation motif induces a conformational change that releases ERK from cytoplasmic anchors and exposes the Ser-Pro-Ser-containing NTS, which is subsequently phosphorylated to enable IPO7 binding and nuclear import. A prototypical NTS was defined in ERK1/2 as a phosphorylatable Ser-Pro-Ser motif (83, 88). In EGR1, phosphorylation of a C-terminal Ser-Pro-Ser motif (aa 482–484) enhances IPO7 interaction (90), although a bipartite NLS (aa 315–430) (111) suggests parallel use of classical import pathways. Consistent with this, both EGR1 and c-Jun show only partial dependence on IPO7 (112), indicating that NTS-mediated import operates alongside classical pathways in a context-dependent manner.

The regulation of IPO7–cargo interactions remains poorly understood. IPO7 binding to EGR1 occurs in both stimulated and unstimulated states, albeit with reduced affinity in phosphorylation-deficient mutants, suggesting additional regulatory inputs. Crosstalk between signaling pathways may further modulate these interactions, as indicated by enhanced IPO7–EGR1 association in the presence of the S1004A mutation in IPO7 (113). Phosphorylation of a Ser-Pro-Ser motif within the NTS of cargo proteins, such as ERK, promotes binding to IPO7, facilitating nuclear import followed by RanGTP-mediated cargo release in the nucleus (88, 114). In the NPC, IPO7 was reported to interact with nucleoporins, including Nup93, to facilitate the nuclear import of ERK1/2 and related regulators (107). In addition, early studies in *Drosophila* linked the IPO7 homolog DIM-7 (moleskin) to ERK nuclear localization (68, 115), and genetic analyses suggested contributions from both IPO7 and importin β1. In yeast, the IPO7 paralog Nmd5 mediates MAPK (HOG1) import independently of importin β1 (116), highlighting species-specific differences. In addition, in neuronal systems, IPO7 binds the CDK5 activator p35 and mediates its nuclear import, thereby preventing its association with CDK5 (101). The NLS of p35 was mapped to a lysine-rich cluster (K61–63) at the N terminus. Although a Ser-Pro-Ser motif is also present in this region, it does not appear to function as an NLS. Additional importins may contribute to p35 nuclear import. In dental pulp cells, LPS stimulation both increases IPO7 abundance and promotes interaction with phosphorylated p38, supporting a role in inflammatory signaling (75, 104). A competitive regulatory mechanism has been proposed in which reduced sirtuin binding to IPO7 is accompanied by enhanced association with phosphorylated p38. Consistent with this model, sirtuins interact with multiple importins in a stimulus-induced manner (95).

#### b. Stress responses and mechanotransduction

Cells constantly respond to changes in their environment, including mechanical forces, extracellular matrix stiffness, cell density, and oxidative stress (Fig. 3*C*). These cues trigger signaling pathways that alter the nuclear localization of transcriptional regulators, with IPO7 emerging as an important mediator of stress-responsive and mechanosensitive nuclear import. IPO7’s involvement in mechanosensitive nuclear transport is downstream of integrin-dependent mechanotransduction and cellular stress signaling. This is mainly mediated by two IPO7-dependent cargoes, the transcriptional regulators YAP1 and RUNX, which undergo regulated nuclear translocation in response to mechanical cues and are key mediators of mechanotransduction (72, 73, 97). IPO7 itself behaves as a mechanoresponsive transport receptor whose localization changes in response to cell density and mechanical stress (97). Binding to YAP1, a central Hippo pathway coactivator regulated by integrin signaling and extracellular matrix interactions, identifies IPO7 as a mediator of mechanotransduction-dependent nuclear import (97, 117). However, an alternative import pathway involving a folded WW domain-based NLS in YAP1, which is recognized by importin α/β1, has also been described (118). Genetic studies in *Drosophila* further support this model: depletion of the IPO7 homolog moleskin reduces Yorkie nuclear localization, suppresses tissue overgrowth, and disrupts integrin-dependent wing development (97, 115). IPO7 also contributes to mesenchymal stem cell differentiation during tooth development by mediating the nuclear import of RUNX transcription factors (72, 73, 74). RUNX proteins regulate transcription in part through interactions with TEAD, which also binds YAP1, linking these factors to cellular responses to mechanical tension (72, 97). Direct binding between IPO7 and RUNX2 has been experimentally demonstrated (73). Finally, IPO7 appears to be limiting under certain conditions, as depletion of YAP1 enhances nuclear accumulation of SMAD3 and ERK2, suggesting competition among cargoes for IPO7 (97).

IPO7 also mediates nuclear import of Forkhead box O (FOXO) transcription factors (119). For example, IPO7–FOXO1 interactions have been implicated in cold stress adaptation in stem cells (65), and inhibition of FOXO1 nuclear export enhances cold tolerance in pre-obese mice. Similarly, IPO7–FOXO3 interactions are modulated by chlorogenic acid treatment (120). There are Ser-Pro-Ser and Ser-Pro-Thr motifs in FOXO1 and FOXO3, but it remains unclear whether these motifs are part of a functional NTS. Related Ser-Pro-Ser-dependent mechanisms have also been implicated in the nuclear localization of Drosha and Notch1. In Drosha, a Ser-Pro-Ser motif directs nuclear import (96), whereas in Notch1, phosphorylation of a Ser-Pro-Ser motif has been linked to glycogen synthase kinase 3β activity, although the involvement of IPO7 remains to be established (92). IPO7 also participates in cellular responses to hypoxia, oxidative stress, and other stress-related pathways (65, 98, 119, 120, 121, 122). HIF-1α is imported through partially redundant pathways involving IPO4, IPO7, and importin β1 (98). Consistent with a role for IPO7 in HIF-1α signaling, reduced IPO7 expression was associated with diminished HIF-1α activity following curcumin treatment, although direct regulation of HIF-1α nuclear import remains unresolved (122).

Interestingly, IPO7 may preferentially bind NLS-containing cargoes under conditions of elevated intracellular cation concentrations (123). Finally, IPO7-mediated transport has been proposed to be directly modulated by the cellular redox state, although it remains unknown whether disulfide-dependent heterodimerization broadly regulates IPO7- (as well as TNPO1- and IPO8)-dependent nuclear transport during oxidative stress (119).

#### c. Hormones

Hormonal signaling controls a wide range of physiological responses through ligand-dependent activation of nuclear receptors and intracellular kinase cascades (Fig. 3*D*). IPO7 contributes to these responses by mediating the stimulus-induced nuclear import of selected receptors and signaling proteins activated by hormones. IPO7 also mediates nuclear transport downstream of hormonal signaling pathways. In contrast to mechanotransduction or cytokine-responsive cargoes, hormone-regulated IPO7 transport frequently involves nuclear receptors or kinase signaling pathways whose localization is controlled by ligand binding, receptor activation, or cellular stress. Early studies demonstrated that IPO7 mediates nuclear transport of the GR in yeast (99, 100, 124). Similarly, in humans, IPO7 mediates GR nuclear import and is impaired by oxidative stress, leading to glucocorticoid insensitivity (99). IPO7 and IPO8 bind GR at residues K513 to 515 in a RanGTP-dependent manner, independent of hormone binding, indicating that receptor availability rather than ligand occupancy governs this interaction (100). An NTS-like element is also found in the NL2 region of the GR, which binds Sxm1 in yeast and IPO7 and IPO8 in mammals (100). These findings suggest that IPO7-mediated transport contributes to stress-responsive regulation of glucocorticoid signaling and may influence transcriptional adaptation during inflammation and oxidative stress.

IPO7 has also been identified as a karyopherin responsible for nuclear import of thyroid hormone receptor α1, although direct binding has not been demonstrated (124). Beyond nuclear receptors, IPO7 also regulates hormone-responsive kinase signaling pathways. More recently, 3-phosphoinositide-dependent kinase-1 has been shown to translocate to the nucleus *via* IPO7 following insulin stimulation, where it phosphorylates AKT and contributes to downstream signaling responses (125). Together, these findings suggest that IPO7 participates in multiple hormone-responsive signaling networks that couple endocrine signals to nuclear regulatory programs.

## Clinical implications

### IPO7 involvement in cancer biology

**Figure 4:**
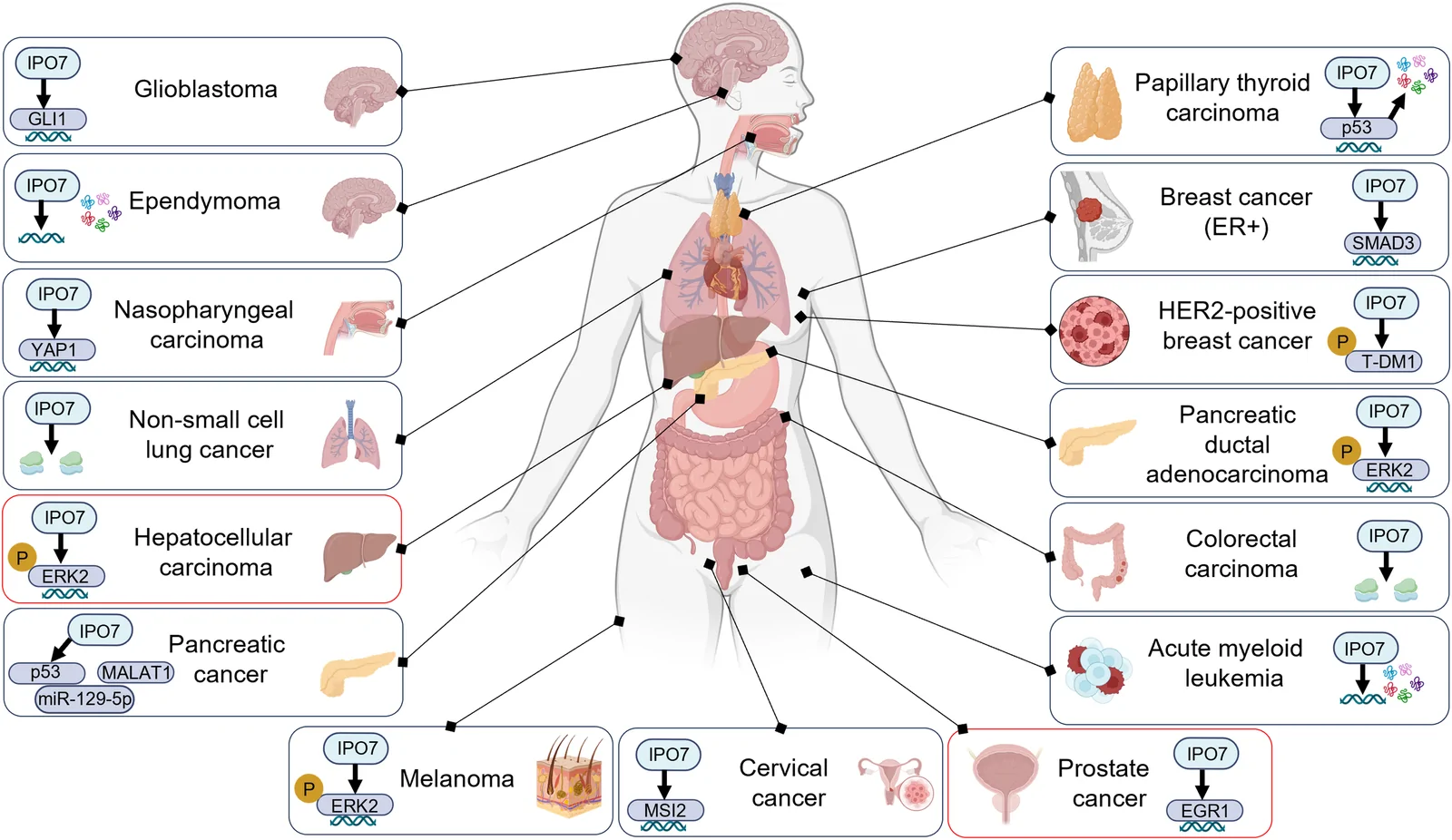
**IPO7 dysregulation in human cancers.** IPO7 has been implicated in numerous human malignancies. The central schematic highlights organs in which IPO7 dysregulation has been reported, whereas the surrounding panels summarize representative cancer types and their major IPO7-dependent cargoes or signaling pathways. *Black frames* indicate dysregulation of IPO7 reported in human tumors and tumor-derived cell lines. *Red frames* indicate findings reported only in experimental model cell lines. The image was created with BioRender.com. AML, acute myeloid leukemia; EGR1, early growth response protein 1; ER+, estrogen receptor-positive; ERK1/2, extracellular signal-regulated kinases 1 and 2; GLI1, GLI family zinc finger 1; HER2, human epidermal growth factor receptor 2 (ERBB2); IPO7, importin 7; MALAT1, metastasis-associated lung adenocarcinoma transcript 1; miR-129-5p, microRNA-129-5p; MSI2, Musashi RNA-binding protein 2; P, phosphorylated; p53, tumor protein p53 (TP53); SMAD3, SMAD family member 3; T-DM1, trastuzumab emtansine (ado-trastuzumab emtansine); YAP1, Yes-associated protein 1.

Several reports implicate IPO7 in multiple aspects of cancer development, progression, and metastasis (Fig. 4). Its role appears to be context-dependent, exhibiting both proapoptotic and antiapoptotic functions depending on the tumor type and cellular environment. Fundamental hallmarks of malignancy, including enhanced proliferation, elevated metabolic demand, and increased reactive oxygen species production, require increased ribosome biogenesis and chromatin assembly, processes that depend on efficient nuclear transport of ribosomal proteins and histones. In this context, IPO7 may contribute to tumorigenesis by supporting biosynthetic capacity and participating in signaling crosstalk.

Initial evidence linking IPO7 to cancer dates back to early observations correlating increased IPO7 expression with colorectal carcinoma proliferation (126). This effect was attributed to enhanced delivery of histone H1 and ribosomal proteins, potentially driven by c-Myc-dependent upregulation. Subsequent studies have revealed a complex interplay between IPO7 and p53 signaling. In papillary thyroid carcinoma, IPO7 overexpression correlates with reduced p53 stability, potentially mediated through miR-7-5p and MDM2-dependent pathways (127). Conversely, p53 has been proposed to negatively regulate IPO7 expression, forming a feedback loop with c-Myc, which promotes IPO7 and XPO1 expression to sustain ribosome biogenesis (79). IPO7 participates in a pro-tumorigenic regulatory axis involving p53, MALAT1, and miR-129-5p in pancreatic cancer, where elevated IPO7 expression correlates with poor clinical outcomes (128, 129). Depletion of IPO7 in these models suppresses tumor growth and metastasis. Additional studies have linked IPO7 to diverse oncogenic signaling pathways. In prostate cancer, treatment with 2′-benzoyloxycinnamaldehyde induces reactive oxygen species production, upregulates EGR1, and enhances the nuclear translocation of signaling components. IPO7 expression increases under these conditions, and its knockdown attenuates the therapeutic response (130). In acute myeloid leukemia, IPO7 is upregulated following FLT3 ligand stimulation, although its functional role remains unexplored (131). In non-small cell lung cancer, IPO7 downregulation following treatment with the herbal formulation Chijongdan has been associated with reduced ribosome biogenesis and increased apoptosis (132).

A recurring theme across cancer models is the involvement of IPO7 in MAPK signaling, particularly through regulation of ERK1/2 nuclear transport (Fig. 4). In melanoma, disruption of the IPO7–ERK interaction using competitive peptides reduces nuclear ERK signaling and inhibits tumor growth (83, 133). Similarly, in hepatocellular carcinoma, MDM2-binding protein interferes with the interaction between phosphorylated ERK1/2 and IPO7, suppressing cell migration and metastasis (85). In pancreatic ductal adenocarcinoma, stabilization of IPO7 mRNA by cingulin enhances nuclear accumulation of phosphorylated ERK and promotes ERK pathway activation (134). IPO7 also contributes to oncogenic transcriptional programs in brain tumors. In glioblastoma, IPO7 mediates nuclear import of GLI1 downstream of FOXM1, forming a FOXM1–IPO7–GLI1 regulatory axis that promotes tumor growth (135).

In breast cancer, IPO7-dependent transport pathways intersect with multiple signaling networks (Fig. 4). For example, FOXA1 reduces the interaction between SMAD3 and IPO7 in estrogen receptor-positive cells (136). In HER2-positive breast cancer, nuclear delivery of the antibody-drug conjugate T-DM1 fused to an NLS was shown to depend on IPO7 but not importin β1, suggesting alternative transport mechanisms even for cNLS-containing cargoes (123). In nasopharyngeal carcinoma, the circular RNA circRILPL1 enhances nuclear accumulation of IPO7 and promotes its interaction with YAP1, thereby increasing YAP1 transcriptional activity (117).

IPO7 expression has also been associated with poor prognosis in multiple tumor types, including cervical cancer and ependymoma (137, 138). Recently, IPO7-dependent nuclear import of MSI2 was linked to cervical cancer progression through enhancement of c-MYC-dependent transcriptional programs and metabolic reprogramming (139). Additional studies have linked IPO7 to nuclear transport of hormone receptors, such as the ubiquitin-specific peptidase 22 associated with the androgen receptor complex (Fig. 3*D*), and to increased association with the minichromosome maintenance complex following DNA damage (78, 140). Collectively, these findings position IPO7 not only as a prognostic and diagnostic marker but also as an active regulator of stimulus-induced nuclear transport in cancer. By modulating the nuclear localization of signaling regulators such as ERK, EGR1, GLI1, and SMAD proteins, IPO7 integrates nucleocytoplasmic transport with oncogenic signaling networks.

### IPO7 involvement in nuclear import of viruses

Many viruses require nuclear access for genome replication or regulation, particularly in nondividing cells, where transport occurs through the NPC (141). Over the past 2 decades, a growing body of evidence has implicated IPO7 in the nuclear trafficking of DNA, viral genomes, and viral proteins.

#### a. Exogenous DNA

**Figure 5:**
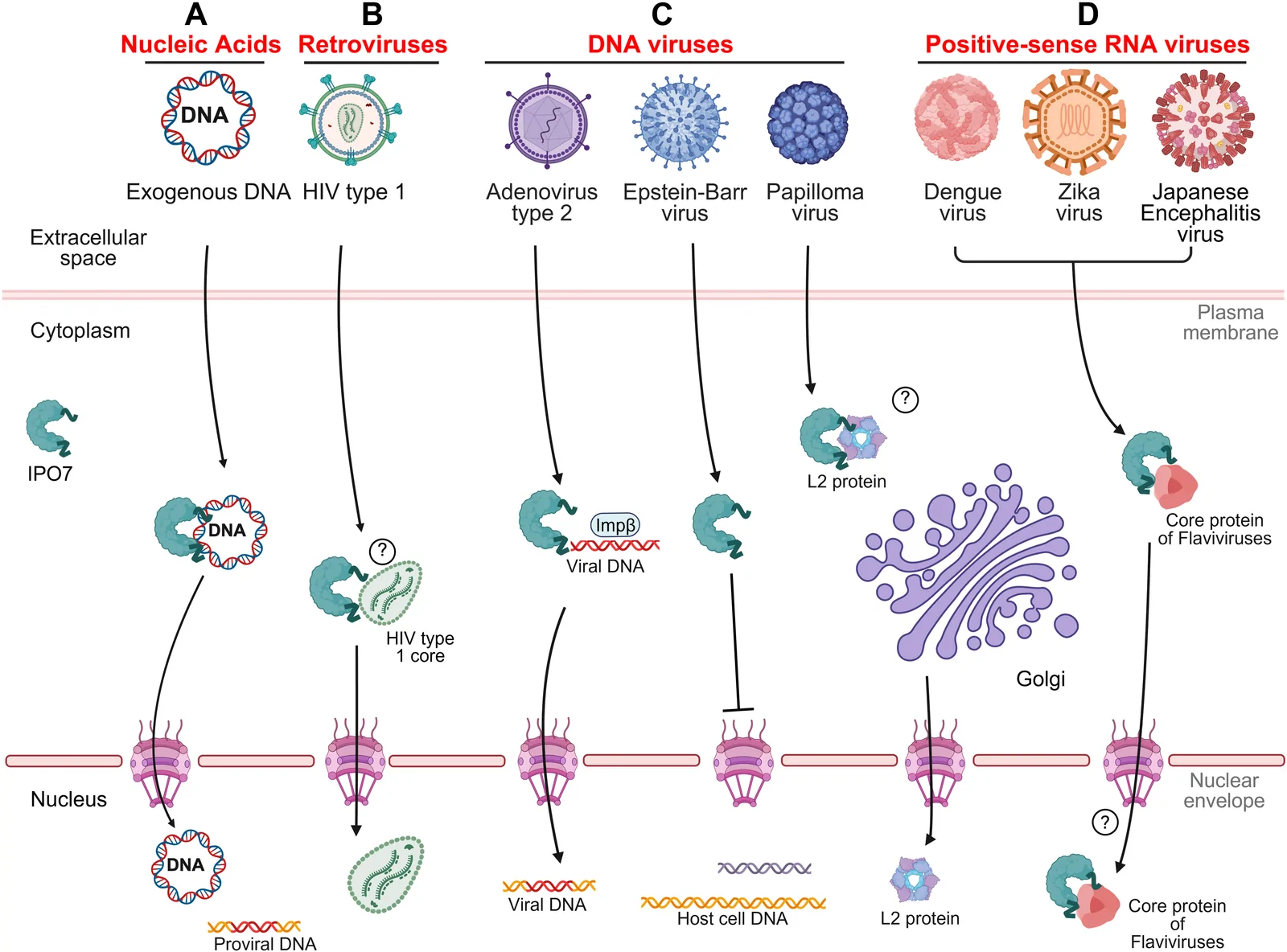
**IPO7-dependent nuclear trafficking pathways exploited by viruses.***A*, IPO7-mediated nuclear import of exogenous nucleic acids. *B*, proposed roles of IPO7 in nuclear trafficking of HIV-1 reverse transcription complexes and viral DNA. *C*, IPO7-dependent nuclear trafficking pathways in DNA viruses, including adenovirus, Epstein-Barr virus, and papillomavirus. *D*, IPO7-mediated nuclear import of flaviviral core proteins from Dengue virus, Zika virus, and Japanese encephalitis virus. *Question marks* indicate transport steps or IPO7 interactions for which the molecular mechanism remains unresolved. Virions and cellular structures are not shown to scale. The image was created with BioRender.com. IPO, importin; HIV, human immunodeficiency virus; Impβ, importin β1; L2, minor capsid protein L2 of papillomavirus.

Early studies reported an interaction between IPO7 and exogenous DNA, exemplified by supercoiled plasmid DNA and human mitochondrial DNA (77) (Fig. 5*A*). These studies suggested that IPO7 may facilitate the nuclear accumulation of exogenous DNA, including mitochondrial DNA, raising the possibility that this import receptor contributes to communication between mitochondria and the nucleus and participates in the activation of innate immune responses. Depletion of IPO7 impaired nuclear accumulation of plasmid and mitochondrial DNA in mammalian cells, whereas supplementation of digitonin-permeabilized cells with IPO7 restored nuclear import (77). These observations led to the hypothesis that viruses may exploit the IPO7 import pathway for nuclear delivery of their genomes. Accordingly, cell lines stably depleted of IPO7 exhibited reduced nuclear import of the human immunodeficiency virus type 1 (HIV-1) DNA and lower transfection efficiency, although IPO7 is not essential for HIV-1 infection (142).

#### b. Retroviruses

IPO7 has been implicated in the nuclear trafficking of the HIV-1 reverse transcription complex and the viral genome (142) (Fig. 5*B*). Similar to ribosomal proteins and histone H1, components of the reverse transcription complex are highly basic, which may facilitate interaction with the acidic surface of IPO7, including its characteristic acidic loop; however, indirect association mediated by nucleic acids cannot be excluded. Pull-down experiments have demonstrated strong binding between IPO7 and HIV-1 integrase (142), which was recently characterized in detail using multiple biophysical approaches (143). Accordingly, knockdown of endogenous IPO7 in HEK293T cells leads to cytoplasmic accumulation of HIV-1 integrase, suggesting a role in nuclear import. IPO7 has been proposed to cooperate with importin β1 in transporting the HIV-1 replication complex, although its interaction with importin β1 appears relatively weak (144). More recent studies suggest that the HIV-1 core can enter the nucleus prior to complete uncoating (145, 146, 147, 148), raising questions about the precise role of IPO7 in this process. Although depletion of IPO7 attenuates infection, it remains unclear whether IPO7 directly engages the viral core.

#### c. DNA viruses

IPO7 has also been implicated in the infection cycles of large DNA viruses (70, 149, 150, 151). In the case of adenovirus type 2, IPO7, together with importin β1, has been proposed to facilitate genome delivery to the nucleus, potentially through interactions with histone H1-bound viral DNA (152) (Fig. 5*C*). Antibody-mediated inhibition of IPO7 and importin β1 reduces viral infection, supporting a functional role for these transport receptors. In cells infected with Epstein–Barr virus, IPO7 expression is modulated by viral microRNAs (70). Specifically, BART3 and BART16 target IPO7 mRNA, thereby reducing its expression and contributing to cellular transformation. These findings highlight a broader interplay between viral factors and the host nuclear transport machinery.

For human papillomavirus (HPV), the viral life cycle includes a critical step in which the viral genome enters the nucleus. Following endocytosis, the cellular process by which extracellular material is internalized into membrane-bound vesicles, HPV DNA, encapsidated by the L1 and L2 proteins, is trafficked to endolysosomal compartments and subsequently to the trans-Golgi network. Nuclear access is thought to occur during mitosis, the process of cell division during which the nuclear envelope breaks down, permitting entry of the viral genome (153). However, the precise mechanisms governing post-Golgi trafficking and nuclear delivery remain incompletely understood. Evidence suggests that HPV particles associate with microtubules and chromatin, facilitating nuclear retention following mitosis. In addition, several nuclear transport receptors, including importin α/β1 and IPO7, have been reported to interact with the L2 protein and/or viral particles (Fig. 5*C*) (153, 154), suggesting that karyopherins could contribute to intracellular trafficking, chromatin targeting, or postmitotic nuclear retention.

#### d. Positive-sense RNA viruses

Members of the Flaviviridae family, including Dengue virus, Zika virus, and Japanese encephalitis virus, encode capsid proteins that localize to the nucleus despite cytoplasmic viral replication (149) (Fig. 5*D*). In this context, IPO7 has been identified as a key mediator of nuclear import of the flaviviral core protein (149). Mutations in the NLS of the Japanese encephalitis virus core protein attenuate infection in mouse models, indicating that nuclear trafficking is functionally important. These findings suggest that IPO7-dependent nuclear import of viral components contributes to viral maturation and/or virion release, suggesting that IPO7-dependent nuclear trafficking may be conserved among flaviviruses.

## Conclusive remarks

The studies reviewed here establish IPO7 as a versatile nuclear transport receptor that participates in both the constitutive nuclear import of housekeeping cargoes, such as ribosomal proteins and histones, and in stimulus-induced transport pathways that couple extracellular signaling to dynamic changes in nuclear composition and gene regulation. Nonetheless, several important technical limitations continue to impede mechanistic dissection of the IPO7 pathway.

*First*, knockdown studies of importin seven have largely relied on siRNA, resulting in reduced expression rather than complete ablation. This leaves open the possibility that residual protein, including potential complexes with IPO8 or other transport receptors, remains functional and contributes to nuclear transport. In addition, the intrinsic biological redundancy between IPO7 and IPO8 complicates loss-of-function analyses and limits our ability to define cargoes for which IPO7 is strictly necessary and sufficient.

*Second*, the specificity of binding interactions detected *in cellulo* may be influenced by IPO7’s tendency to copurify with nucleic acids, potentially leading to indirect associations or hetero-complex formation with other importins or cellular factors. Similarly, the limited quality and sensitivity of available antibodies may contribute to variable or misleading results.

*Third*, unlike well-established transport signals such as cNLSs, PY-NLSs, and IBB/beta-like import receptor binding domains, the proposed NTSs recognized by IPO7 lack a defined consensus sequence that can be reliably identified from primary sequence alone, making cargo prediction substantially less straightforward.

*Fourth*, recombinant IPO7 produced in bacteria lacks posttranslational modifications (*e.g.*, SUMOylation, phosphorylation, and acetylation) that are likely important for its activation and function. This may explain the relatively low binding affinity and specificity for cargoes reported in several studies (80), particularly those using human IPO7.

*Finally*, the absence of high-resolution structural information has limited the development of structure-guided biochemical approaches and hindered mechanistic understanding of the IPO7 pathway to a level comparable to that achieved for other β-karyopherins, including importin β1, Kapβ2, and CRM1.

Future studies should systematically reevaluate IPO7 cargoes to establish specificity and dependence on posttranslational modifications. High-resolution structural information will be essential to define the molecular basis of cargo recognition. In parallel, the development of tissue-specific *in vivo* models will be critical to elucidate IPO7 function across different cellular contexts. Finally, identifying the extracellular stimuli and upstream receptors that regulate IPO7 activation remains an important goal. From a physiological standpoint, the identification of IPO7 as a mechanosensitive import receptor for YAP1 suggests potential roles in embryonic heart development, cardiac homeostasis, mechanotransduction, and stress adaptation, processes in which YAP1 signaling plays a central role.

## Conflict of interest

The authors declare that they have no conflicts of interest with the contents of this article.
